# Mechanisms and Functions of Long Non-Coding RNAs at Multiple Regulatory Levels

**DOI:** 10.3390/ijms20225573

**Published:** 2019-11-08

**Authors:** Xiaopei Zhang, Wei Wang, Weidong Zhu, Jie Dong, Yingying Cheng, Zujun Yin, Fafu Shen

**Affiliations:** 1 State Key Laboratory of Crop Biology, College of Agronomy, Shandong Agricultural University, NO. 61 Daizong Street, Tai’an 271018, Shandong, China; xpzhang@sdau.edu.cn (X.Z.); weiwang@sdau.edu.cn (W.W.); 2018110136@sdau.edu.cn (J.D.); 2017110205@sdau.edu.cn (Y.C.); 2 State Key Laboratory of Cotton Biology, Chinese Academy of Agricultural Sciences Cotton Research Institute, Key Laboratory for Cotton Genetic Improvement, Anyang 45500, Henan, China; zwdd1005@gmail.com

**Keywords:** lncRNA, epigenetic, transcriptional, post-transcriptional, translation, post-translational modification

## Abstract

Long non-coding (lnc) RNAs are non-coding RNAs longer than 200 nt. lncRNAs primarily interact with mRNA, DNA, protein, and miRNA and consequently regulate gene expression at the epigenetic, transcriptional, post-transcriptional, translational, and post-translational levels in a variety of ways. They play important roles in biological processes such as chromatin remodeling, transcriptional activation, transcriptional interference, RNA processing, and mRNA translation. lncRNAs have important functions in plant growth and development; biotic and abiotic stress responses; and in regulation of cell differentiation, the cell cycle, and the occurrence of many diseases in humans and animals. In this review, we summarize the functions and mechanisms of lncRNAs in plants, humans, and animals at different regulatory levels.

## 1. Introduction

Long non-coding (lnc) RNA transcripts are longer than 200 nt in length and have no protein-coding ability; however, they may have short open reading frames (ORFs). Most annotated lncRNAs are RNA polymerase II (Pol II) transcribed; hence, they are similar in structure to mRNA and may have cap structures and poly A tails. In addition, the plant-specific RNA polymerases, Pol IV and V, can produce lncRNAs [1,2]. LncRNAs comprise a diversified class of long intergenic non-coding (linc)-RNAs, long non-coding antisense transcripts, long intronic non-coding RNAs, and non-overlapping antisense lncRNAs [3]. LncRNA expression levels are lower than those of mRNA, and their sequence conservation is poor. For these reasons, few studies have been conducted on lncRNAs in plants, and this field remains in its infancy. In recent years, with the rapid development of biotechnology, many lncRNAs have been identified in animals and plants through microarrays, tiling arrays, expressed sequence tags (ESTs), and RNA-Seq. Many animal lncRNA biological functions have been confirmed; however, the biological functions of only a few plant lncRNAs have been studied [4,5]. Studies have indicated that lncRNAs are involved in epigenetic, transcriptional, post-transcriptional, and translation regulation, as well as post-translational modification [6,7,8]. LncRNAs have various regulatory functions in humans and animals. The main regulatory roles at the chromatin level are dosage compensation effects, genomic imprinting, chromatin modification, and remodeling [9,10,11]. LncRNAs also participate in regulating alternative splicing, cell differentiation, and cell cycle regulation, and they are involved in regulating the occurrence of many diseases [12,13,14,15]. Plant lncRNAs play key roles in flowering time, root organogenesis, seedling photomorphogenesis, sexual re-production, crop yield, and responses to biotic and abiotic stresses [16,17,18,19].

**Figure 1:**
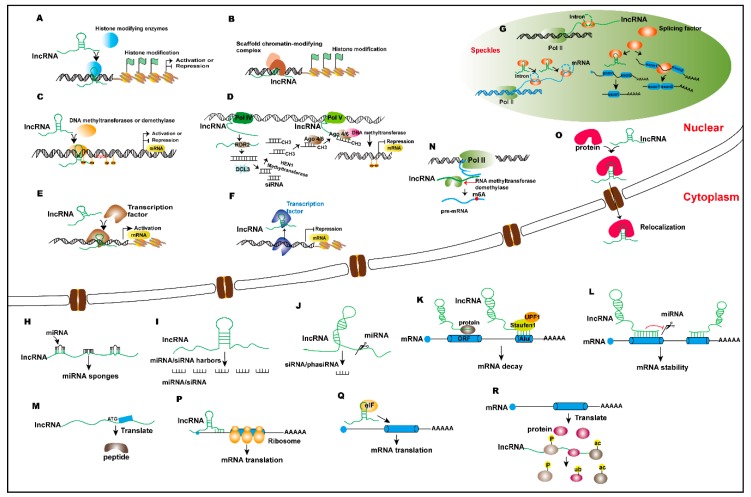
Regulatory mechanisms of lncRNAs at the genome level. (**A**) lncRNAs interact with histone-modifying enzymes to activate or repress gene transcription. (**B**) lncRNAs recruit histone-modified complexes or act as scaffolds for multiple histone modifiers to regulate histone modification of genes and thereby regulate gene transcription. (**C**) lncRNAs recruit DNA methyltransferases or demethylases to regulate the target gene transcription. (**D**) Pol IV/V transcribed lncRNAs are involved in RNA-dependent DNA methylation, thus activating or repressing gene transcription. (**E**,**F**) lncRNAs interact with transcription factors to activate or repress gene expression. (**G**) lncRNAs interact with splicing factors or proteins to regulate the mRNA alternative splicing; splicing factors directly regulate the lncRNA’s alternative splicing in speckles. (**H**) lncRNAs act as miRNA sponges that regulate target gene expression. (**I**) lncRNAs act as miRNA or small interfering RNAs (siRNA) precursors. (**J**) miRNAs target lncRNAs to produce siRNA or phased small-interfering RNAs (phasiRNAs). (**K**) lncRNAs are involved in the Staufen1-mediated mRNA decay, and lncRNAs bind to proteins and mediate mRNA decay. (**L**) lncRNAs directly bind to mRNA and regulate mRNA stability, or competitively bind to mRNA to improve mRNA stability. (**M**) lncRNAs can be translated to peptides. (**N**) lncRNAs interact with RNA methyltransferases or demethylases and thus regulate mRNA expression. (**O**) lncRNAs combine with proteins to regulate protein localization. (**P**) lncRNAs interact with mRNAs and affect mRNA translation. (**Q**) lncRNAs bind the translation initiation complex eIF (eukaryotic initiation factor) to regulate mRNA translation. (**R**) lncRNAs interact with proteins and control protein phosphorylation, acetylation, and ubiquitination at the post-translation level.

According to the current understanding of lncRNA identification and function, these transcripts are broadly classified into those mediating chromatin modifications and DNA methylation involved in epigenetic regulation, proteins (primarily transcription factor) and DNA interactions involved in transcriptional level regulation, and in mRNA processing at the post-transcriptional level, as well as direct interactions with proteins to regulate protein translation and post-translation modification (Figure 1). In this review, we discuss the biological functions and regulatory mechanisms of some plant and human lncRNAs.

## 2. lncRNAs Are Involved in Regulating Histone Modifications at the Chromatin Level

Epigenetics refers to heritable alterations in gene expression without alterations in DNA sequence. The molecular mechanisms involved primarily include DNA methylation, histone modification, chromatin remodeling, and, in a broad sense, non-coding RNA. Histone modifications are a major class of chromatin modifications responsible for the epigenetic regulation of gene expression. Histones undergo various modification processes such as methylation, acetylation, phosphorylation, and ubiquitination under the action of related enzymes, and different modifications are associated with activation or inhibition of gene expression. Histone methylation and acetylation are the most important histone modifications, and they occur primarily at histone H3 lysine residues. In response to a variety of diseases and plant flowering, histone modification can induce changes in chromatin structure, thus affecting transcriptional regulation [20,21,22]. lncRNAs are a new class of epigenetic regulators that play important roles in epigenetic regulation. lncRNAs regulate epigenetic modification primarily in the nucleus, regulating gene transcription at the transcriptional level by modulating histone or DNA modification, primarily methylation and acetylation. lncRNAs can regulate histone methylation or acetylation alone [23], or can act as a scaffold that interacts with enzymes or methylation and acetylation complexes, thereby regulating histone methylation and acetylation simultaneously [24]. Here, we discuss how several lncRNAs regulate gene expression through interacting with histone-associated acetylase and methylase and recruiting chromatin modification complexes at the chromatin level.

### 2.1. lncRNAs Interact with Histone-Modified Complexes or Enzymes to Regulate Histone Methylation

**Figure 2:**
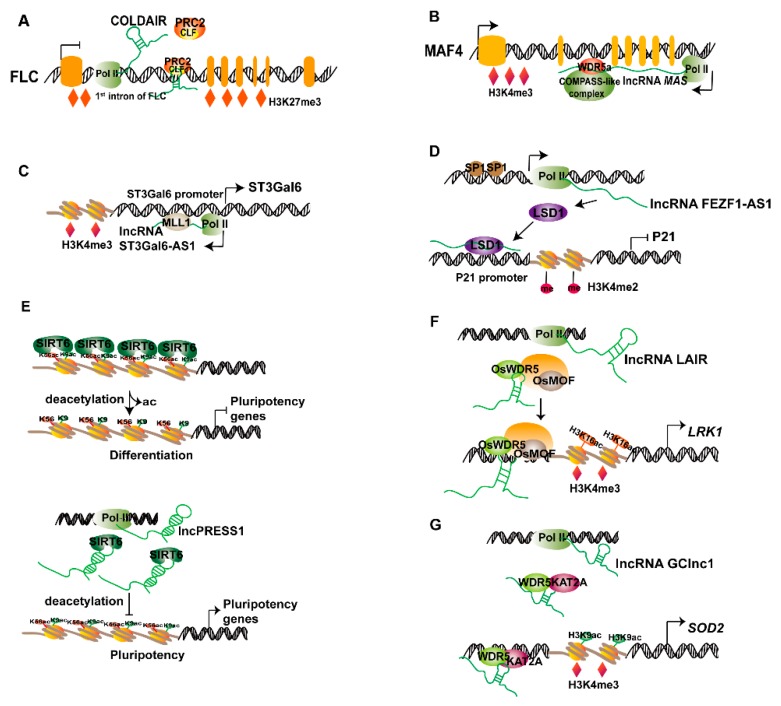
lncRNAs regulate histone methylation and acetylation to mediate gene transcription at the chromatin level. (**A**) lncRNA COLD ASSISTED INTRONIC NONCODING RNA (*COLDAIR*) is transcribed from the first intron of FLOWERING LOCUS C (*FLC*). *COLDAIR* mainly binds to CURLY LEAF (CLF) components in the polycomb repressive complex 2 (PRC2) complex, and the PRC2 complex is then recruited to the *FLC* chromatin and mediates trimethylation of H3K27, thereby establishing *FLC* silencing during vernalization. (**B**) lncRNA *MAS* binds to WDR5a and recruits it to MADS AFFECTING FLOWERING4 (*MAF4*) sites, thus facilitating H3K4me3 and activating *MAF4* expression. (**C**) lncRNA ST3Gal6 antisense 1 (ST3Gal6-AS1) binds MLL1, recruits it to the promoter region of ST3Gal6, and induces H3K4me3 to activate ST3Gal6 transcription. (**D**) The transcription factor SP1 binds the promoter of lncRNA *FEZF1-AS1* and accelerates *FEZF1-AS1* expression; *FEZF1-AS1* recruits lysine-specific demethylase 1 (LSD1) to the P21 promoter to induce H3K4me2 demethylation and inhibit P21 transcription. (**E**) SIRT6 enhances H3K56/H3K9 deacetylation, thus repressing pluripotency gene expression; *lncPRESS1* binds to SIRT6 and inhibits its deacetylation activity, thus activating pluripotency gene expression. (**F**) lncRNA *LAIR* recruits OsMOF and OsWDR5a, colocalizes at the *LRK1* chromatin region, increases the abundance of H3K4me3 and H3K16ac, and activates *LRK1* transcription. (**G**) lncRNA gastric cancer-associated lncRNA 1 (GClnc1) acts as a scaffold for KAT2A and WDR5 and recruits them to the superoxide dismutase 2 (*SOD2*) promoter, thus increasing H3K4me3 and H3K9ac levels and *SOD2* transcription. ‘→’ indicates facilitation and activation, ‘⟞’ indicates repression.

Histone methylation has been shown to be involved in many biological processes in animals and plants. Histone methylation has complex functions; for example, H3K4, H3K36, and H3K79 promote gene transcription, whereas H3K9, H3K27, and H4K20 inhibit gene transcription [20,25]. Changes in histone methylation at lysine sites are regulated by histone methyltransferases or demethylases (including absent, small, or homeotic-1 (ASH1), mixed Lineage Leukemia 1 (MLL1), enhancer of Zeste homolog 2 (EZH2), and lysine-specific demethylase 1 (LSD1)) [26]. lncRNAs regulate histone methylation, thereby affecting gene transcription, primarily in two ways: by regulating histone methyltransferases or demethylases or by recruiting chromatin-modifying complexes (such as polycomb repressive complex 2 (PRC2) [27] and COMPASS-like complex [28]) to the chromatin region (Figure 1A). The first discovered plant lncRNA was found to regulate histone methylation in the chromatin region of FLOWERING LOCUS C (*FLC*), encoding an important flowering inhibitor protein, thus regulating the vernalization in *Arabidopsis*. The lncRNA transcribed from *FLC* intron 1, consistent with the direction of *FLC* transcription, has been named COLD ASSISTED INTRONIC NONCODING RNA (*COLDAIR*) [22]. It is an intronic lncRNA with a 5’ cap structure but no polyadenylation site and is transcribed by RNA polymerase II (RNAPII); its expression peaks after 20 days of cold exposure. After knock down of *COLDAIR* through RNA interference, *FLC* expression decreases, and transgenic lines show late flowering after vernalization. In vitro RNA binding assays have confirmed the direct interaction between *COLDAIR* and the CXC domain of CURLY LEAF (CLF), which is a component of PRC2 and is homologous to Enhancer of Zeste (a histone methyltransferase), and mediates trimethylation of H3K27. *COLDAIR* primarily participates in the recruitment of PRC2 to the *FLC* chromatin under cold conditions, thus promoting H3K27me3 accumulation and establishing *FLC* gene silencing during vernalization (Figure 2A) [22,29]. In addition to *COLDAIR*, the lncRNA *MAS* has recently been discovered to regulate the expression of MADS AFFECTING FLOWERING4 (*MAF4*), which fine-tunes the time of flowering. The lncRNA *MAS* is a *MAF4* cognate antisense transcript that contains polyadenylation site and localizes to the nucleus. The 5’-end of *MAS* initiates at the transcriptional termination site of *MAF4*, whereas the 3’-end of *MAS* extends to intron 1 of *MAF4* [30]. *MAS* is induced by cold conditions and positively regulates *MAF4* expression and represses flowering. Further studies have shown that *MAS* does not regulate *MAF4* through a sRNA mechanism, nor does it regulate the stability of the *MAF4* transcript; instead, it *cis*-regulates *MAF4* expression at the transcriptional level. Chromatin immunoprecipitation (ChIP) has been used to detect the accumulation of the histone activation marker H3K4me3 at *MAF4* loci in Col-0 (WT) and *amiR-MAS-1/2* (*MAS* amiRNA knockdown) lines, thus indicating that it is regulated by *MAS*. Experiments have shown that the deposition of H3K4me3 and activation of *MAF4* require WD40 containing repeat 5a (WDR5a), a core component of the COMPASS-like complexes [28]. Chromatin isolation by RNA purification (ChIRP)-qPCR and RNA immunoprecipitation (RIP) assays have indicated that *MAS* binds WDR5a and recruits it to *MAF4* sites, thereby enhancing H3K4me3 and activating *MAF4* expression (Figure 2B) [30].

lncRNAs regulate histone methylation by recruiting histone-modified complexes or by directly interacting with histone methyltransferases or demethylase to regulate gene transcription. The lncRNA ST3Gal6 antisense 1 (ST3Gal6-AS1) is transcribed from the promoter region of ST3Gal6 in the opposite direction from transcription. The lncRNAs ST3Gal6-AS1 and ST3Gal6 are down-regulated in colorectal cancer (CRC), and their expressions levels are positively correlated—lncRNA ST3Gal6-AS1 activates the expression of ST3Gal6 in CRC cells. ChIP has been used to detect the H3K4me3 signal in the promoter region of ST3Gal6, and knockout of ST3Gal6-AS1 affects the accumulation of H3K4me3. Bioinformatics analysis and RIP experiments have indicated that lncRNA ST3Gal6-AS1 binds histone methyltransferase MLL1, recruits it to the promoter region of ST3Gal6, and induces H3K4me3 modification, which in turn activates ST3Gal6 transcription (Figure 2C) [31]. Furthermore, the highly expressed lincRNA *FEZF1-AS1* has been identified in gastric cancer. High expression of *FEZF1-AS1* increases the sizes of gastric cancer tumors in patients and also promotes the proliferation of gastric cancer cells by inducing cell cycle progression and by decreasing apoptosis. The lincRNA *FEZF1-AS1* inhibits P21 expression in the cell cycle. RNA pull-down and RIP experiments have confirmed that the lincRNA *FEZF1-AS1* specifically binds lysine-specific demethylase 1(LSD1), thereby regulating the expression of P21 at the transcriptional level. ChIP assays have confirmed that LSD1 directly binds the promoter of P21, thereby mediating H3K4me2 demethylation modification; knocking out *FEZF1-AS1* affects the binding of LSD1 to the P21 promoter and H3K4me2 modification. The transcription factor SP1 upregulates *FEZF1-AS1* expression, and ChIP assays have indicated that SP1 binds the promoter of *FEZF1-AS1*. The lincRNA *FEZF1-AS1* recruits LSD1 to the promoter region of P21, thereby inducing demethylation of H3K4me2 and inhibiting the transcription of P21 (Figure 2D) [32].

### 2.2. lncRNAs Interact with Histone-Modified Complexes or Enzymes to Regulate Histone Acetylation

Histone acetylation is a reversible dynamic equilibrium process catalyzed by histone acetyltransferase (HAT) and histone deacetylase (HDAC). Histone acetylation refers to the activation of gene transcription by HAT through acetylation of histone lysine residues, whereas HDAC catalyzes histone deacetylation and inhibits gene transcription. The acetylation sites of histones are primarily H3K4Ac, H3K9Ac, H3K27Ac, H3K56Ac, and H4K16Ac. The mechanism through which lncRNAs regulate histone acetylation modification is similar to the mechanism of regulation of histone methylation modification mentioned above. lncRNAs regulate histone acetyltransferases or deacetylases (such as HDAC, HAT, CREB-binding protein (CBP/P300), males absent on the first (MOF), K acetyltransferases 2A (KAT2A), and sirtuin 6 (SIRT6) [33]) and thus activate or inhibit gene transcription (Figure 1A).

Jain et al. [23] have used RNA-seq and ChIP-seq techniques to identify transcriptome and histone post-transcriptional modifications and p53 binding regulation in human embryonic stem cells (hESCs) undergoing differentiation, thus defining a high-confidence set of 40 lncRNAs, which are regulated by p53 binding to promoters during hESC differentiation, and whose expression is regulated by epigenetic regulation of chromatin status. Because these lncRNAs are regulated by p53 and are ESC-specific, they have been named *lncPRESS*. *lncPRESS1* is one of 40 multi-exonic intergenic lncRNAs, and is 756 nt in length. By using gene silencing technology to interfere with p53, *lncPRESS1* has been found to regulate the transcription of p53, which keeps the pluripotency of hESC consistent with the core pluripotency factor. RNA-seq techniques have been used to sequence hESCs lacking *lncPRESS1*, and *lncPRESS1* has been found to control the gene network promoting pluripotency. RNA pull-down assays in vitro have indicated that *lncPRESS1* binds SIRT6, an H3K56ac/K9ac histone deacetylase [34]. The deletion of *lncPRESS1* enhances SIRT6-mediated deacetylation of H3K56/K9ac in pluripotency genes. *lncPRESS1* mediates the activity of SIRT6 and inhibits the deacetylation of SIRT6 on pluripotency genes by binding SIRT6, thus maintaining the hESC state (Figure 2E).

### 2.3. lncRNAs Act as Scaffold Regulating Histone Methylation and Acetylation

In addition to regulating histone methylation or acetylation alone, lncRNAs act as molecular scaffolds that bind two or more protein molecules that subsequently perform specific biological functions (Figure 1B). Yang et al. [19] have identified the lncRNA *LAIR* from the antisense transcript of *LRK* (leucine-rich repeat receptor kinase) gene clusters and found that *LRK1* is located in the first intron of *LAIR*. *LAIR* has a 5’cap structure and polyadenylation site and as many as 10 alternative spliced isoforms; splicing events have been detected in the second exon. Overexpression of *LAIR* has been found to affect rice plant growth and grain yield. Antisense lncRNA can regulate the expression of the corresponding sense strand coding gene and neighboring protein-coding genes [35]. Sense *LAIR* significantly increases the expression of *LRK1* and *LRK4* transcripts and activates their promoter, but has no effect on the expression of other *LRK* genes. ChIP-qRT-PCR results have indicated that overexpression of *LAIR* increases the abundance of H3K4me3 and H3K16ac at *LRK1* chromatin. Further experiments have indicated that *LAIR* binds the chromatin-modifying proteins OsMOF and OsWDR5 and co-localizes at the *LRK1* genomic locus, thus activating *LRK1* transcription and increasing grain yield (Figure 2F) [19]. In a recent study, Sun et al. [24] identified gastric cancer-associated lncRNA 1 (GClnc1) whose expression is positively correlated with pathological differentiation, vascular invasion, tumor size, and the American Joint Committee on Cancer (AJCC) stage. RNA pull-down experiments have indicated that WDR5 and KAT2A specifically bind GClnc1. WDR5 is an H3K4 methyltransferase, and KAT2A is an H3K9 acetyltransferase. RIP experiments have demonstrated that GClnc1 binds WDR5 and KAT2A, thus forming a complex. Knocking down GClnc1 decreases the binding ability of WDR5 and KAT2A in the whole genome and inhibits the expression of related genes; the promoter regions of these genes show correspondingly lessened levels of H3K4me3 and H3K9ac. Superoxide dismutase 2 (*SOD2*) is one of the target genes of the WDR/KAT2A complex, and GClnc1 regulates its expression. ChIP assays have demonstrated that WDR5 and KAT2A directly bind the *SOD2* promoter and consequently dynamically regulate H3K9ac and H3K4me3 in the *SOD2* promoter region. Therefore, GClnc1 can act as a scaffold molecule that recruits the WDR5 and KAT2A complex to the promoter of *SOD2*, thereby regulating histone modification and activating transcription of the *SOD2* gene (Figure 2G).

## 3. lncRNAs Are Involved in Regulating DNA Methylation at the DNA Level

DNA methylation is an important epigenetic modification that maintains genomic integrity and regulates gene expression. DNA methylation does not change the sequence and composition of nucleotides, but gene expression is affected. Methylation modifications are catalyzed by different DNA methylases, but most occur in CpG (cytosine-phosphate diester-guanine) islands in the gene promoter region, and methylation of the gene promoter region can result in transcriptional silencing that suppresses gene expression. The mechanism of DNA methylation in plants is more complex than that in mammals. DNA methylation in plants is primarily accomplished by the silencing of gene transcription induced by RNA interference. Plant-specific RNA polymerases IV and V transcribe lncRNAs and participate in RNA-dependent DNA methylation (RdDM) [36]. In mammals, lncRNAs recruit DNA methyltransferases to the promoters of target genes, thus resulting in methylation of the corresponding target gene of lncRNAs.

### 3.1. lncRNA Are Involved in RdDM

In plants, RdDM is an epigenetic modification process that primarily relies on two core proteins: Dicer-like 3 (DCL3), which cleaves long double-stranded RNA to produce small interfering RNAs (siRNAs), and Argonaute 4 (AGO4), which binds siRNAs to function [37,38]. Simultaneously, the transcriptional mechanism of RdDM primarily depends on two plant-specific RNA polymerases: Pol IV and Pol V [2,39]. Plant-specific RNA Pol IV and Pol V can produce lncRNAs, and RNA Pol IV-transcribed lncRNAs are cleaved into 24 nt siRNAs by DCL3. These siRNAs are methylated and bind AGO protein, thus forming AGO-siRNA complex. RNA Pol V-transcribed lncRNAs act as scaffold molecules that recruit AGO-siRNA complexes through sequence complementarity as well as DNA methyltransferase (DOMAINS REARRANGED METHYLTRANSFERASE 2 (DRM2) and CHROMOMETHYLASE3 CMT3), which mediates de novo methylation, thereby resulting in gene silencing (Figure 1D) [1,36,40]. Here, we introduce two plant lncRNAs that use RdDM as to regulate gene transcription.

**Figure 3:**
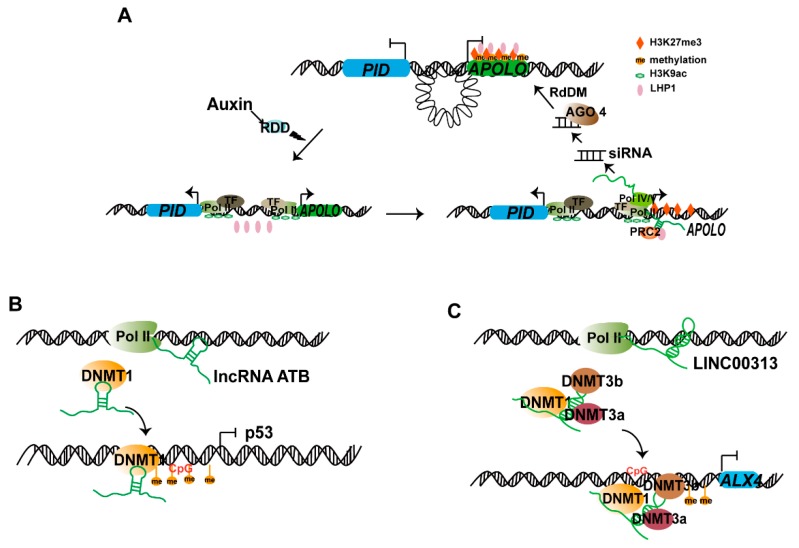
lncRNAs regulate DNA methylation to control gene transcription and expression at the DNA level. (**A**) lncRNA AUXIN REGULATED PROMOTER LOOP RNA (*APOLO*) controls auxin-dependent chromatin loop dynamics in the *PID* promoter region. In response to auxin, RDD (ROS1, DML2, and DML3)-mediated *APOLO* DNA demethylation is activated, the chromatin loop opens, and levels of the activated mark H3K9ac increase and H3K27me3 levels decrease, thus increasing *APOLO* and *PID* expression. Subsequently, RNA Pol II-transcribed *APOLO* recruits LHP1 and PRC2 to redeposit repressive H3K27me3 in the *PID* and *APOLO* promoters, whereas Pol IV/V-transcribed *APOLO* transcripts trigger DNA methylation, thus facilitating the formation of a chromatin loop and downregulation of *PID* expression. (**B**) lncRNA ATB recruits DNA methyltransferase (DNMT1) to the p53 promoter and facilitates p53 methylation, thereby inhibiting p53 expression. (**C**) LINC00313 binds to DNMT1 and DNA methyltransferase (DNMT3b) and recruit them to the aristaless-like homeobox 4 (ALX4) promoter, thus facilitating the methylation of ALX4 promoter and inhibiting ALX4 expression. PSMS: photoperiod-sensitive male sterility; ‘→’ indicates facilitation and activation, ‘⟞’ indicates repression.

The *lncRNA* AUXIN REGULATED PROMOTER LOOP RNA (*APOLO*) [41] is a lincRNA located 5148 bp upstream of the *PINOID* (*PID)* gene and is transcribed by RNA Pol V and II. The *APOLO* locus transcribed by RNA Pol V is highly methylated [42]. *PID* is induced by the APOLO gene, and *APOLO* and *PID* are co-expressed under endogenous auxin treatment. Further analysis has revealed that *APOLO* transcribed by RNA Pol II or plant-specific Pol IV/V polymerase may dynamically regulate the formation of chromatin loops in the *PID* promoter, thereby controlling *PID* gene expression. Auxin activates RDD (repressor of silencing 1 (ROS1), DEMETER-LIKE proteins DML2, and DML3)-mediated DNA demethylation in the gene region where *APOLO* is originally transcribed. The chromatin loop gradually opens, the activated histone modification H3K9ac increases, and the inhibitory modified H3K27me3 decreases. Transcripts of *APOLO* and *PID* are synthesized by RNA Pol II and accumulate. RNA Pol Ⅱ-transcribed *APOLO* transcript binds the PRC1 components LHP1 (Like Heterochromatin Protein-1) and PRC2, and the repressive marks H3K27me3 are redeposited at the promoters of *PID* and *APOLO*. The Pol V-synthesized *APOLO* transcript produces a 24 nt siRNA and recruits the AGO4 complex, which in turn favors DNA methylation. Consequently, *APOLO* silencing is mediated via RdDM. Pol II *APOLO*-LHP1 complex and Pol IV/V-dependent DNA methylation directly control the dynamic chromatin loop changes in the promoter region of *PID* and regulate the expression of the *PID* gene and subsequently the response to auxin (Figure 3A) [42].

### 3.2. lncRNAs Regulate DNA Methylation by Interacting with DNA Methyltransferase

DNA methylation in genomes is catalyzed by DNA methyltransferases and is regarded as a key player in epigenetic silencing of transcription. The members of the DNA methyltransferase (DNMT) family are conventionally classified as de novo (DNMT3a-b) and maintenance (DNMT1) methyltransferases. lncRNAs can recruit DNA methyltransferases to regulate target gene transcription (Figure 1C). lncRNAs have been shown to cause abnormal methylation of target genes by binding DNMT1 methyltransferase. The long noncoding RNA activated by TGF-β (lncRNA ATB) is highly expressed in renal cell carcinoma; it facilitates proliferative and migratory abilities and inhibits apoptosis of renal cell carcinoma cells. The lncRNA ATB inhibits expression of p53, a well-known tumor-suppressor gene. DNMT1 positively regulates the methylation level of p53 and participates in the occurrence of various tumors [43]. RIP and ChIP assays have indicated that the lncRNA ATB binds DNMT1, and DNMT1 binds the p53 promoter. Thus, lncRNA ATB recruit DNMT1 to the p53 promoter and facilitate p53 methylation, thereby inhibiting p53 expression (Figure 3B) [44].

Additionally, researchers have found that a class of lncRNAs recruit multiple DNMTs (DNMT1, DNMT3a, and DNMT3b) to target genes, thereby leading to methylation of target genes and regulating their expression. LINC00313 is highly expressed in thyroid cancer and negatively regulates aristaless-like homeobox 4 (ALX4) expression. Dual-luciferase reporter gene assays have indicated that LINC00313 binds the ALX4 promoter, which has many CpG islands and is easily methylated. RIP and ChIP assays have revealed that LINC00313 binds DNMT1 and DNMT3B, which in turn bind the ALX4 promoter. These results indicate that LINC00313 recruit DNMT1 and DNMT3b and consequently facilitate the methylation of the ALX4 promoter and inhibit ALX4 expression, thereby regulating cell proliferation, migration, and invasion in thyroid cancer (Figure 3C) [45].

## 4. lncRNAs Involved in the Process of Transcriptional Regulation

lncRNAs also directly participate in the regulation of transcription itself, a process that primarily occurs in the nucleus. lncRNAs participate in regulation of transcription in many ways. lncRNAs directly bind DNA sequences and inhibit gene transcription; in addition, they directly interact with proteins (primarily transcription factors) and inhibit or activate the expression of downstream genes (Figure 1E,F). Here, we discuss the mechanisms and functions of lncRNAs involved in transcriptional regulation.

### 4.1. lncRNAs Function in Transcription Activation

**Figure 4:**
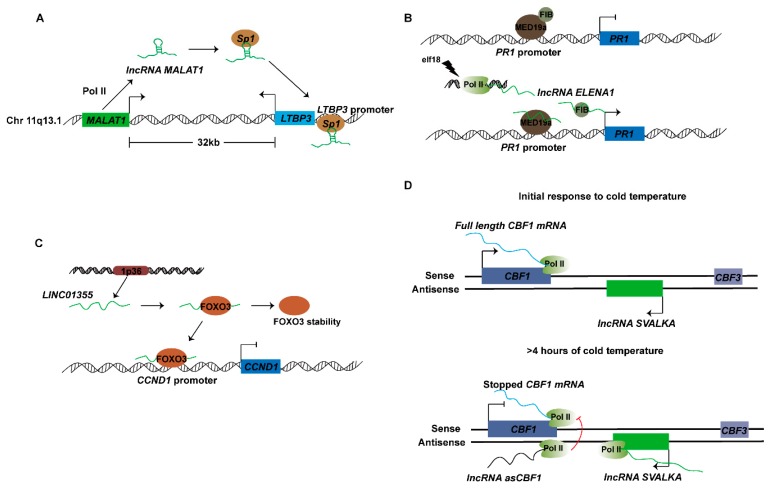
Regulatory mechanism of lncRNAs at the transcriptional level. (**A**) lncRNA *MALAT1* (metastasis-associated lung adenocarcinoma transcript 1) and *LTBP3* (latent transforming growth factor-*β* (TGF-*β*)-binding proteins) mRNA are located on the same chromosome 11q13.1; *MALAT1* directly promotes *LTBP3* transcription by recruiting the transcription factor Sp1 to the *LTBP3* promoter. (**B**) Mediator subunit 19a (MED19a) binds the PATHOGENESIS-RELATED GENE 1 (*PR1*) promoter, and FIBRILLARIN 2 (*FIB2*) directly interacts with MED19a and represses *PR1* expression before elf18 treatment. After elf18 treatment, lncRNA ELF18-INDUCED LONG-NONCODING RNA1 (ELENA1) transcript level increases, and ELENA1 directly binds to MED19a and *FIB2*, thus blocking binding of *FIB2* to MED19a and promoting the release of FIB2 from the PR1 promoter to enhance PR1 expression. (**C**) LINC01355 interacts with the transcriptional repressor forkhead box O3 (FOXO3), promotes its stability, and recruits it to the CCND1 promoter, thus repressing CCND1 transcription. **(D)** lncRNA *SVALKA* (*SVK*) represses sense *CBF1* transcription. Initially during cold exposure, *SVK* is not expressed, and sense *CBF1* is normally transcribed. After 4 h of cold exposure, lncRNA *SVK* read-through transcription results in the expression of antisense *CBF1* lncRNA (*asCBF1*) and increased RNAP II occupancy on both strands. The transcription of lncRNA *asCBF1* results in RNAP II collision, thus restricting the expression of full-length CBF1. ‘→’ indicates facilitation and activation, ‘⟞’ indicates repression.

lncRNAs can act as co-factors that modulate transcription factor activity (Figure 1E). For example, the mouse lncRNA *Evf2* is transcribed from an ultraconserved distal enhancer, which specifically binds the transcription factor DLX2, forming a transcriptional complex, and binds an enhancer, thereby inducing expression of the adjacent protein-coding gene DLX5/6 [46]. The lncRNA *MALAT1* (metastasis-associated lung adenocarcinoma transcript 1) is expressed in several cancers and is highly conserved in mammals. The *MALAT1* and *LTBP3* (latent transforming growth factor-*β* (TGF-*β*)-binding proteins) genes are located on chromosome 11q13.1, and *MALAT1* is located approximately 32.0 kb upstream of the *LTBP3* gene, in the opposite from transcription. *LTBP3* plays an important role in the activation of TGF-*β* and regulates the bioavailability of TGF-*β*, especially in bone. *MALAT1* and *LTBP3* have been found to be highly expressed in mesenchymal stem cell (MSCs) from all 25 patient samples relative to that from healthy donors examined. Knockdown of *MALAT1* inhibits *LTBP3* transcription and TGF-*β* secretion in MSCs from the bone marrow of myeloma patients, whereas overexpression of *MALAT1* increases *LTBP3* transcription and TGF-*β* secretion in MSCs from healthy donors. These results indicate that *LTBP3* transcription is dependent on *MALAT1*. *MALAT1* cooperates with the transcription factor Sp1 in increasing *LTBP3* transcription in MSCs. ChIP assays have shown that Sp1 directly interacts with the core element of the *LTBP3* promoter in MSCs. Knockout of *MALAT1* inhibits the abundance of Sp1 at the *LTBP3* promoter. In conclusion, the lncRNA *MALAT1* directly promotes transcription of the *LTBP3* by recruiting Sp1 transcript factor to *LTBP3* promoter (Figure 4A) [47].

Plant immune responses are initiated by the recognition of conserved pathogen-associated molecular patterns by plant pathogens or pattern recognition receptors. lncRNAs have been reported to participate in response to plant immunity. Seo et al. have identified a long intergenic noncoding RNA associated with the immune response in *Arabidopsis*, designated ELF18-INDUCED LONG-NONCODING RNA1 (ELENA1), as a positive factor enhancing resistance against *Pseudomonas syringe pv tomato DC3000*. ELENA1 is an intergenic lncRNA 589 nt in length. Its expression is induced by both elf18 and flg22 treatment. Knockout and overexpression of ELENA1 in plants affects the expression of PATHOGENESIS-RELATED GENE 1 (*PR1*) after elf18 treatment and responds to pathogen resistance. RNA-seq analysis of plants overexpressing ELENA1 after elf18 treatment has confirmed that the expression of defense-related genes is higher than that in WT. ELENA1 directly interacts with the mediator subunit 19a (MED19a), thus affecting the enrichment of MED19a on the *PR1* promoter, and ELENA1 and MED19a together induce *PR1* expression [48]. Recently, ELENA1 has also been found to interact with FIBRILLARIN 2 (*FIB2*), which in turn directly interacts with MED19a. *FIB2* is a negative transcriptional regulator of *PR1* immune responsive gene expression. Further analysis has indicated that ELENA1 blocks the binding of *FIB2* to MED19a, promotes the release of *FIB2* from *PR1* promoter, and enhances the expression of *PR1* (Figure 4B) [49].

### 4.2. lncRNAs Function in Transcriptional Interference

lncRNAs also have inhibitory effect on the transcriptional regulation of genes and can cooperate with transcription factors and complexes to repress gene transcription and also regulate RNAP II activity through other mechanisms (Figure 1F). For example, the lncRNA LINC01355 is transcribed from the 1p36 locus in humans and is a tumor suppressor in breast cancer—LINC01355 inhibits cyclin D1 (CCND1) expression. RNA pull-down and RIP assays have shown that LINC01355 interacts with the transcription factor forkhead box O3 (FOXO3) and facilitates the stability of FOXO3 protein. FOXO3 is a transcriptional repressor of CCND1 [50]. ChIP assays have demonstrated that overexpression of LINC01355 results in selective enrichment of FOXO3 protein in the CCND1 promoter region. LINC01355-repressed CCND1 expression depends on FOXO3-mediated transcriptional regulation (Figure 4C) [51].

Plants respond to changing environmental conditions by regulating gene expression. Early events in cold response in *Arabidopsis* include rapid transcriptional upregulation of intron-less C-repeat/dehydration-responsive element binding factor (CBF), a gene encoding a highly conserved transcription factor that promotes cold tolerance in many plant species, which is usually arranged in a single cluster [52]. Recently, through the transcription start site sequencing method, after *Arabidopsis* growth at 22 and 4 °C for 3 h, a cold-induced lncRNA was identified on the antisense strand between *CBF3* and *CBF1*, and was named *SVALKA* (*SVK*) [53]. *SVK* is transcribed by Pol II and is involved in regulating endogenous *CBF1* expression. Furthermore, Kindgren et al. have constructed three informative T-DNA insertion mutants. The results of these mutant studies have indicated that *SVK* inhibits cold-induced *CBF1* expression and has biological effects on cold acclimation and cold tolerance in *Arabidopsis*. Antisense transcripts of *CBF1* (*asCBF1*) have been detected in the *hen2-2* mutant enzymes. The 3’-UTR of the *CBF1* gene is responsible for degrading non-coding Pol II transcripts, and the transcription of *asCBF1* is dependent on *SVK*. Further studies have shown that lncRNA *SVK* read-through transcription results in the expression of antisense *CBF1* lncRNA (*asCBF1*). The transcription of lncRNA *asCBF1* results in Pol II collision, thus restricting the expression of full-length *CBF1*, in a cascade regulatory model initiated by *SVK* (Figure 4D). This study demonstrates the important roles of lncRNA in cold acclimation and provides a precise regulatory mechanism [53].

## 5. lncRNAs Are Involved in Post-Transcriptional Regulation

In addition to the two aforementioned mechanisms, lncRNAs also directly participate in the post-transcriptional regulation of mRNAs, primarily in the processing of mRNAs, including alternative splicing, RNA editing, protein translation, and transport, which are important for gene functional diversification. lncRNAs can also interact with miRNAs to subsequently regulate target gene expression in multiple biological processes (Figure 1G–O). Here, we summarize some lncRNAs involved in post-transcriptional regulation.

### 5.1. lncRNAs and Alternative Splicing

Alternative splicing (AS) is an important mechanism for spatial and temporal regulation of gene expression and for proteomic diversity. Most of the genes (~95%) in human cells are selectively spliced [54]. Many RNA-binding proteins and splicing factors are involved in the splicing regulation process, including splicing factors with serine/arginine (SR) structural proteins with RNA recognition domains or heterogeneous nuclear ribonucleoprotein (hnRNP) family proteins [55], as well as other proteins and splicing factors containing RNA-binding domains [56]. These splicing factors or proteins can interact with lncRNA and subsequently regulate mRNA alternative splicing, and splicing factors can also directly regulate lncRNA alternative splicing (Figure 1G).

**Figure 5:**
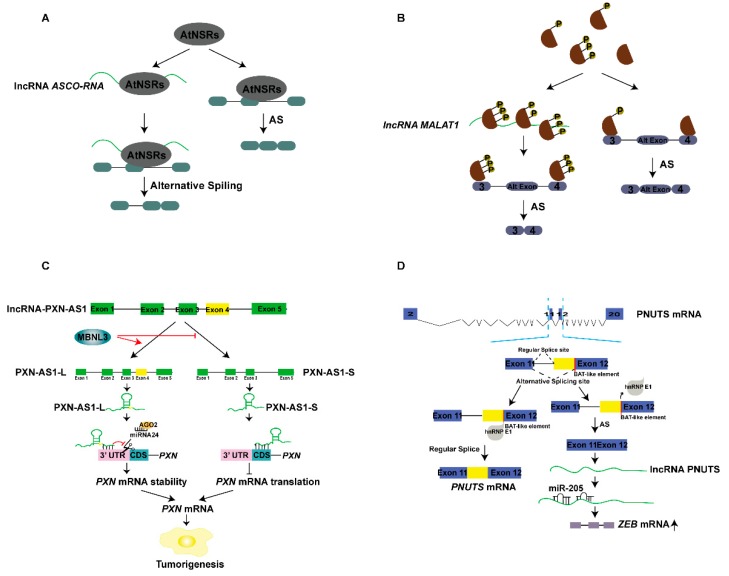
lncRNAs regulate mRNA alternative splicing and alternative splicing of lncRNAs. (**A**) lncRNA alternative splicing competitor RNA (ASCO-RNA) competitively binds to *Arabidopsis* AtNSRs with mRNA and regulates alternative splicing (AS) in auxin signaling. (**B**) lncRNA MALAT1 affects the localization and distribution of serine/arginine-rich (SR) proteins in nuclear speckles and the phosphorylation of SR proteins, and recruits SR proteins to pre-mRNAs, thereby regulating AS. (**C**) The splicing factor muscleblind-like-3 (MBNL3) facilitates lncRNA PXN-AS1-L (containing exon 4) transcript expression and inhibits PXN-AS1-S (deleting exon 4); PXN-AS1-L promotes the stability of *PXN* mRNA by competing with the miRNA-24-AGO2 complex for binding to the 3’-UTR region of *PXN* mRNA. PXN-AS1-S represses *PXN* mRNA translation through directly binding to the CDS region of *PXN* mRNA. MBNL3 regulates lncRNA PXN-AS1 AS, thus affecting *PXN* mRNA expression in hepatocellular carcinoma (HCC) tumorigenesis. (**D**) PNUTS mRNA has an AS site and a BAT-like element in exon 12. Heterogeneous nuclear ribonucleoprotein (hnRNP) E1 binds the BAT element and promotes PNUTS pre-RNA splicing, thus producing PNUTS mRNA. HnRNP E1 removal from BAT alternative splicing sites allows PNUTS pre-RNA AS to produce lncRNA-PNUTS. lncRNA PNUTS acts as miR-205 sponge promoting expression of the target gene ZEB.

lncRNAs are involved in alternative splicing mechanisms. ENOD40 and lnc351 [41], renamed alternative splicing competitor RNA (ASCO-RNA), interact with nuclear selective splicing regulator nuclear speckle RNA-binding proteins (NSRs) and subsequently regulate gene alternative splicing. NSRs are a family of RNA-binding proteins (RBPs) that act as nuclear AS regulators in *Arabidopsis*. NSR1 relocates from the nucleus to the cytoplasm in root tissues where ENOD40 is expressed, whereas ASCO-RNA does not. The role of AtNSR is associated with primary and lateral root (LR) meristem, whose formation and expression patterns are regulated by the key phytohormone auxin. Lnc351 (ASCO-RNA) is induced by auxin, and AtNSRs regulate AS and bind specific target mRNAs. ASCO-RNA competitively binds NSRs with mRNA and interferes with the alternative splicing of downstream auxin-responsive genes by NSR protein, thus affecting the growth of lateral roots (Figure 5A) [57]. The lncRNA MALAT1 primarily localizes to nuclear speckles. Human and mouse MALAT1 have an RNA binding site for SR protein splicing factor 1 (SRSF1), and RIP experiments have indicated that MALAT1 binds a subset of the SR proteins, and that SRSF1 protein specifically binds directly to the 5’ region of MALAT1. MALAT1 affects the localization and distribution of SR family proteins in nuclear speckles, as well as the distribution and the ratio of phosphorylated to dephosphorylated pools of SR proteins. MALAT1 recruits SR proteins to the transcriptional active region, thereby regulating the variable cleavage of mRNA precursors (Figure 5B) [12].

There are multiple subtypes of lncRNAs in existence; consequently, lncRNAs have multiple regulatory mechanisms and biological functions. Muscleblind-like-3 (MBNL3) is an oncofetal splicing factor expressed in hepatocellular carcinoma (HCC) tissues. Transcriptome sequencing of SMMC-7721 cells with stable deletion of MBNL3 has identified AS events, among which lncRNA-PXN-AS1 has been found to be involved. LncRNA-PXN-AS1 has five exons, and PXN mRNA locates on its antisense strand. lncRNA-PXN-AS1 has two main transcripts, PXN-AS1-L (containing exon 4) and PXN-AS1-S (lacking exon 4), and MBNL3 upregulates PXN-AS1-L and decreases PXN-AS1-S expression. PXN-AS1-L is highly expressed in HCC cells inconsistent with MBNL3. Both PXN-AS1-L and PXN-AS1-S are primarily located in the cytoplasm, but they have different regulatory effects on PXN. RIP experiments have shown that PXN-AS1-L and PXN-AS2-S interact with *PXN* mRNA. PXN-AS1-L upregulates the PXN protein, whereas PXN-AS1-S downregulates it. PXN-AS1-L upregulates *PXN* mRNA and protein by competing with the miRNA-24-AGO2 complex for binding the 3’-UTR region of *PXN* mRNA, thereby reducing the degradation of PXN induced by the miRNA-24-AGO2 complex, and promoting the stability of PXN mRNA, rather than directly participating in *PXN* mRNA translation. PXN-AS1-S downregulates PXN protein primarily through directly binding the CDS (coding sequence) region of *PXN* mRNA, thus preventing the binding of translation elongation factors to *PXN* mRNA and inhibiting the translation elongation of *PXN* mRNA. The different regulation of *PXN* mRNA by PXN-AS1-L and PXN-AS1-S is primarily due to the inclusion or skipping of exon 4, an AS process regulated by MBNL3. lncRNA-PXN-AS1 and PXN mRNA together execute the role of MBNL3 in HCC tumorigenesis (Figure 5C) [58].

HnRNP E1 is an RNA binding protein and a known alternative splicing (AS) inhibitor [59]. Affymetrix array analysis using an hnRNP E1 knockout-induced epithelial-mesenchymal transition (EMT) model of NMuMG (normal murine mammary gland) cells has shown that PNUTS pre-RNA levels are inhibited, and lncRNA-PNUTS expression is upregulated. The PNUTS gene has an AS site in exon 12, which contains a BAT-like element. HnRNP E1 binds the BAT element and thus inhibits PNUTS pre-RNA splicing to produce PNUTS mRNA. HnRNP E1 removal from BAT AS sites promotes PNUTS pre-RNA AS, thus producing lncRNA-PNUTS, which localizes to the cytoplasm and nucleus. LncRNA-PNUTS can adsorb miR-205 after entering the cytoplasm, thereby promoting the expression of the miR-205 target gene ZEB and consequently regulating the migration and invasion in EMT as well as tumor implantation, growth, and metastasis. PNUTS pre-RNA produces PNUTS mRNA and lncRNA-PNUTS through hnRNP E1-regulated AS, which have different biological functions (Figure 5D) [60].

### 5.2. lncRNA, miRNAs, and siRNAs

#### 5.2.1. lncRNAs Act as miRNA Sponges

miRNAs are a class of non-coding short-sequence RNAs that are 18 to 25 nt in length, do not have an ORF, and are widely expressed in eukaryotes. MiRNAs play important roles in animals and plants by targeting mRNA for cleavage or directing translational inhibition to negatively regulate mRNA expression [61]. miRNAs are an important link in the function of lncRNAs, and the mutual regulation of miRNA and lncRNA is involved in many biological processes. The function of many lncRNAs may be regulation of gene expression by sequestering miRNAs. LncRNAs can act as endogenous target mimics (eTMs), which regulate gene expression by competing with miRNAs (Figure 1H). This mode of action is called “miRNA sponging”, and lncRNAs with this function are called competitive endogenous RNAs (ceRNAs) [62]. The functional study of lncRNAs as endogenous competitive mimic targets of miRNAs is well known, and this is a relatively simple regulatory mechanism among lncRNAs. There are several special databases (such as starBase v2.0, miRsponge, and PeTMbase) related to this mechanism [63,64,65], which allow researchers to select interesting lncRNAs and miRNAs according to the data and study the mechanism of ceRNA interaction. Although the study of ceRNA mechanism is a major direction that most researchers consider in the study of lncRNA mechanism, there are still researchers who believe that the mechanism of ceRNA action is controversial. Denzler et al. found that changes in ceRNA are unlikely to affect lower targets of miRNAs expressed at lower levels; miRNA levels matter only in so much as the miRNAs must reach levels sufficient to inhibit target mRNA. Under normal homeostatic conditions, ceRNA-mediated gene regulation is unlikely to occur [66,67].

**Figure 6:**
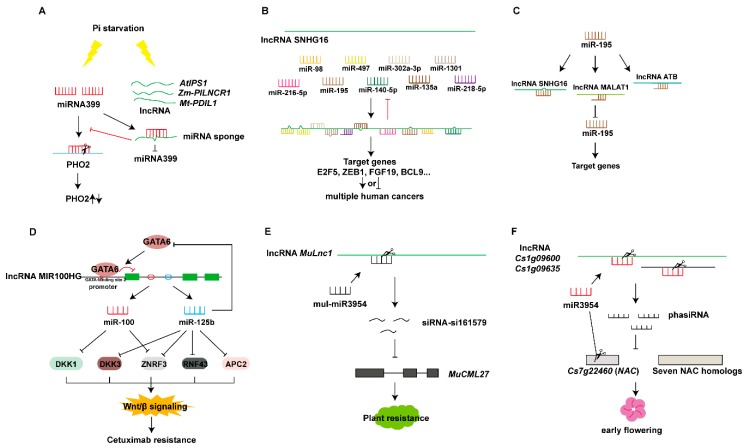
Mutual regulatory mechanisms among lncRNAs and miRNAs. (**A**) Pi starvation induces lncRNA *IPS1*, Pi-deficiency-induced long-noncoding RNA1 (*PILNCR1*), and *PDIL1* levels in *Arabidopsis*, maize, and *Medicago truncatula*, respectively, and these lncRNAs sequester miR399 and block its function in cleavage of *PHO2* transcripts. (**B**) LncRNA small nucleolar RNA host gene 16 (SNHG16) acts as a sponge for many miRNAs (such as miR-98, miR-216-5p, miR-302a-3p, miR-497, miR-140-5p, miR-1301, miR-218-5p, miR-195, and miR-135a), thus regulating gene expression involved in multiple human cancers. (**C**) lncRNA ATB, MALAT1, and SNHG16 function as miR195 sponges regulating target gene expression. (**D**) lncRNA MIR100HG has miR-100 and miR-125b precursor sequences that can produce miR-100 and miR-125b. miR-100 and miR-125b coordinately downregulate five negative regulatory factors (DKK1, DKK3, ZNRF3, RNF43, and APC2) in canonical Wnt/β-catenin signaling, thus increasing Wnt signaling and cetuximab resistance. GATA6 inhibits lncRNA MIR100HG expression, whereas miR-125b targets GATA6 and relieves this repression. (**E**) *MuLnc1* is cleaved by mul-miR3954 and produces the secondary siRNA si161579, which targets and silences calmodulin-like protein gene *CML27* (*MuCML27*) expression. The regulatory network of mul-miR3954-*MuLnc1*-si161579-*MuCML27* is involved in the mulberry response to environmental stress. (**F**) miR3954 directly cleaves two lncRNAs (*Cs1 g09600* and *Cs1 g09635*) and produces phasiRNA, which targets several NAC homologous genes including *Cs7 g22460* (NAC); *Cs7 g22460* is also a miR3954 target gene. The miR3954-lncRNA-phasiRNA-NAC pathway regulates early flowering in citrus. PGMS and TGMS: photoperiod- and thermo-sensitive genic male sterility; ‘→’ indicates facilitation and activation, ‘⟞’ indicates repression.

The lncRNA *IPS1*, which belongs to the *TPSI1/Mt4* family, was first identified in 2007 as having a ceRNA regulatory mechanism. *IPS1* accumulates in leaves and roots under high phosphate starvation. The 23 nt conserved region of the *IPS1* sequence is complementary to the partial sequence of miR399. *IPS1* is not cleaved by miR399, as a bulge is formed by a mismatch at the 10–11 base position of the miRNA; consequently, *IPS1* can mimic miR399 target genes and bind miR399 without being degraded, thereby inhibiting the activity of miR399, increasing the expression of the target gene *PHO2*, and maintaining the normal growth of *Arabidopsis* under phosphate starvation conditions [68]. Recent studies have found that the lncRNA Pi-deficiency-induced long-noncoding RNA1 (*PILNCR1*) in maize has the same mechanism of action as *IPS1*. *PILNCR1* can also be used as a miR399 mimicking target to bind miR399, thereby increasing the accumulation of the target gene PHO2 mRNA and responding to low phosphate stress in maize [69]. Additionally, Zhang et al. have found that *PDIL1* competitively inhibit *MtPHO2* degradation as a target mimic of miR399, thereby regulating Pi transport in *Medicago truncatula* (Figure 6A) [70]. The tomato lncRNA23468 acts as a decoy that adsorbs miR482b for targeted cleavage of miR482b, thereby increasing the accumulation of the miR482b target gene *NBS-LRR* in tomato plants infected with *P. infestans* [71].

Studies on lncRNAs as mimic targets of miRNAs in animals can be conducted more quickly than those in plants. Certain lncRNAs can be found to be mimic targets of multiple miRNAs in animals. Examples of this type of interaction include the lncRNA small nucleolar RNA host gene 16 (SNHG16) and intramuscular fat-associated long non-coding RNA (IMFNCR). The lncRNA SNHG16 functions as an oncogene in multiple cancers and acts as sponge for many miRNAs (such as miR-98, miR-216-5p, miR-302a-3p, miR-497, miR-140-5p, miR-1301, miR-218-5p, miR-195, and miR-135a), thereby regulating expression of genes involved in multiple human cancers (Figure 6B), such as breast cancer [72], cervical cancer [73], HCC [74,75], papillary thyroid cancer [76], retinoblastoma [77], bladder cancer [78], osteosarcoma [79], pancreatic cancer [80], and gastric cancer [81]. The lncRNA IMFNCR competitively binds miR-128-3p and miR-27b-3p, thus decreasing miRNA-targeted cleavage of the target gene peroxisome proliferator-activated receptor-gamma (PPARG) and increasing expression of PPARG, and promotes intramuscular adipocyte differentiation [82]. Similarly, a single miRNA may also act on multiple lncRNAs. Both lncRNA small nucleolar RNA host gene 7 [83] and LINC00662 [84] function as a miR-34a sponge, thereby regulating target gene expression. In addition to binding lncRNA SNHG16, miR195 interacts with the lncRNAs ATB [85] and MALAT1 [86] and promotes target gene expression (Figure 6C).

#### 5.2.2. lncRNAs Act as Precursors of miRNAs And siRNAs

Certain lncRNAs can form precursors of miRNAs through intracellular shearing, and RNAs can be processed to produce specific miRNAs regulating the expression of target genes (Figure 1I). Cai and Cullen et al. first discovered in 2007 that the non-coding RNA H19 produces a 23 nt miRNA precursor of miRNA675 that suppresses translation of insulin growth factor receptor (Igf1r), thereby inhibiting cell proliferation in response to cellular stress or oncogenic signals [87,88]. In recent years, studies on this function of lncRNAs have gradually increased. Lu et al. conducted full exon sequencing, full transcriptional sequencing, and small RNA sequencing of cetuximab-resistant and untreated CRC cells, finding that the lncRNAs MIR100HG, miR-100, and miR-125b are up-regulated in cetuximab-resistant cells. Sequence analysis revealed sequences of miR-100 and miR-125b in lncRNA MIR100HG, thus indicating that it hosts genes of miR-100 and miR-125b. The expression of these three genes is simultaneously up-regulated in different cetuximab-resistant cells. Subsequently, after overexpression of miR-100, miR-125b, and the lncRNA MIR100HG, cetuximab resistance has been obtained in CRC cells and in head and neck squamous cell carcinoma cell lines. The combination of miR-100 and miR-125b maximizes the ability to mediate cancer cell resistance, thus demonstrating the synergistic resistance of the two miRNAs. miR-100 and miR-125b coordinately downregulate five negative regulatory factors (DKK1, DKK3, ZNRF3, RNF43, and APC2) involved in the canonical Wnt/β-catenin signaling pathways (following Wnt signaling), thus increasing Wnt signaling. Further analysis revealed that the lncRNA MIR100HG promoter region has a GATA-binding site 2, and ChIP and electrophoretic mobility shift assay (EMSA) experiments have demonstrated that GATA6 binds the GATA-binding site 2. GATA6 primarily inhibits the expression of the lncRNA MIR100HG, whereas miR-125b targets the 3’-UTR of GATA6, thereby inhibiting its expression and forming a double negative regulatory network in response to cetuximab resistance (Figure 6D) [89].

#### 5.2.3. miRNAs Target lncRNAs to Produce Small RNAs

In addition to lncRNAs functioning as mimic targets of miRNAs, miRNAs can also directly or indirectly regulate lncRNAs. lncRNAs can directly act as targets of miRNAs and can be negatively regulated by miRNAs. miRNAs targeting lncRNAs can produce siRNA or phased small-interfering RNAs (phasiRNAs), thereby regulating gene expression (Figure 1J). PhasiRNAs are a special class of small RNAs that are produced at 21 or 24 nt intervals from transcripts of precursor RNA and are derived from non-coding transcripts; they function in trans in targeting downstream genes [90].

The novel lncRNA *MuLnc1* is expressed at a relatively high level in ripe fruits and is cleaved by mul-miR3954, thus producing secondary siRNAs in 21 nt intervals in mulberry. Analysis of mulberry sRNA libraries has resulted in the identification of abundant 21 nt siRNAs, among which siRNA si16157 can target and silence the expression of the calmodulin-like protein gene *CML27* (*MuCML27*) in mulberry, which plays significant roles in mediating plant stress tolerance. Thus, the regulatory network of mul-miR3954-*MuLnc1*-si161579-*MuCML27* is involved in the mulberry response to environmental stress (Figure 6E). In addition to si161579, the siRNAs derived from *MuLnc1* have also been predicted to target many protein-coding genes with different functions. Therefore, as a precursor of these siRNAs, the lncRNA MuLnc1 may be involved in the response to multiple biotic and abiotic stresses [91].

Citrus miR3954 is specifically expressed in flowers, and miR3954 can target one NAC transcription factor (*Cs7 g22460*) and two lncRNAs (*Cs1 g09600* and *Cs1 g09635*). A phasiRNA has been detected from the miR3954-targeted transcript, which has experimentally been shown to be derived from two lncRNAs. The phasiRNA targets several NAC homologous genes including *Cs7 g22460* (NAC), a miR3954 target gene. Overexpression miR3954 lines show a decrease in lncRNA transcription, an increase in phasiRNA abundance, and a decrease in NAC gene expression, including that of *Cs7 g22460*, thus resulting in early flowering of citrus (Figure 6F). However, homologous genes of these two lncRNAs have not been found in other plants, thus suggesting that this miR3954-lncRNA-phasiRNA-NAC pathway may be citrus specific [92].

### 5.3. lncRNAs Mediate RNA Decay

**Figure 7:**
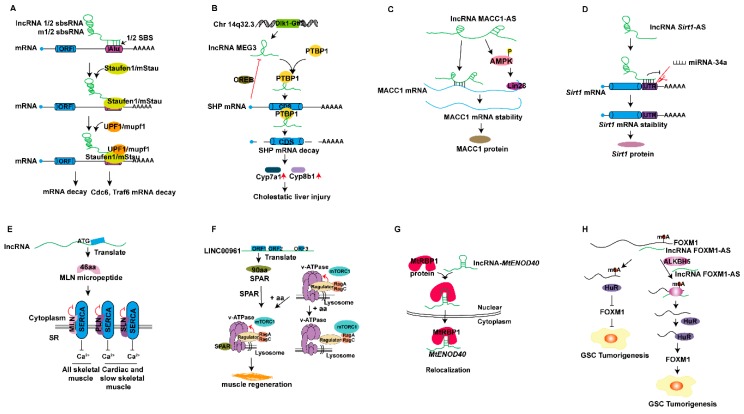
lncRNAs are involved in regulating mRNA decay, mRNA stability, coding polypeptides, protein relocalization, and RNA methylation modification of mRNAs. (**A**) lncRNA 1/2-sbsRNA binds to the Alu element of the 3’UTR region in target gene and forms a functional Staufen 1 (STAU1)-binding site (SBS) that triggers STAU1-mediated mRNA decay (SMD). STAU1 binds to mRNA in a manner dependent on SBS sites, recruits UPF1 protein to STAU1, and leads to the degradation of mRNA in humans. The mouse lncRNA m1/2-sbsRNA also uses the same mechanism. (**B**) lncRNA maternal expression gene 3 (MEG3) interacts with polypyrimidine tract-binding protein 1 (PTBP1) protein and recruits it to the CDS region of small heterodimer partner (SHP) mRNA, thus promoting SHP mRNA decay and activating the expression of target genes *Cyp7a1* and *Cyp8b1*. SHP inhibits the expression of MEG3 by cAMP response element-binding protein-dependent feedback regulation. (**C**) lncRNA MACC1-AS1 is the MACC1 antisense transcript. MACC1-AS1 directly binds MACC1 through a binding site and increases its stability and expression. AMPK activation and subsequent Lin28 translocation play synergistic roles in regulating MACC1 mRNA stability. (**D**) *Sirt1* AS lncRNA interacts with Sirt1 mRNA, thus forming an RNA duplex and improving the stability of *Sirt1* and Sirt1 protein through competition with miRNA-34a in the 3’-UTR of *Sirt1* mRNA. (**E**) lncRNA encodes the 46aa micropeptide myoregulin (MLN), whose function is similar to that of PLN and SLN, through acting directly on sarcoplasmic reticulum Ca2^+^-ATPase (SERCA) on the sarcoplasmic reticulum and preventing Ca2^+^ from entering the SR. (**F**) LINC00961 encodes the polypeptide small regulatory polypeptide of amino acid response (SPAR). The v-ATPase complex interacts with the Ragulator complex in lysosomes and regulates the activation of mTORC1 under amino acid stimulation. SPAR binds to the v-ATPase and the Rags and Ragulator complexes, thus affecting the activity of mTORC1 and inhibiting the recruitment of mTORC1 to lysosomes under amino acid stimulation, thereby promoting muscle regeneration. (**G**) *MtENOD40* interacts with MtNSR1 protein, and MtNSR1 is re-localized from nuclear speckles to the cytoplasm in Medicago. (**H**) lncRNA FOXM1-AS is opposite from the transcription direction of FOXM1 and complementary to the last exon of FOXM1 mRNA. FOXM1-AS cooperates with ALKBH5 in regulating FOXM1 nascent transcript expression through promoting binding of HuR to FOXM1, thereby promoting glioblastoma stem-like cells (GSC) tumorigenesis. SR: sarcoplasmic reticulum; ‘→’ indicates facilitation and activation, ‘⟞’ indicates repression.

The abundance of mRNA directly affects the production of protein. In cells, the abundance of mRNA is usually indicated by two aspects: the amount of transcription and the rate of decay. lncRNAs can mediate the decay of mRNA (Figure 1K). A Staufen 1 (STAU1)-binding site (SBS) can be formed by incomplete base pairing between the Alu element in the 3’-UTR of an Staufen 1 (STAU1)-mediated mRNA decay (SMD) target and the Alu element in another polyadenylation non-coding RNA in the cytoplasm. These lncRNAs are termed half-Staufen 1 (STAU1)-binding site RNAs (1/2-sbsRNAs). The lncRNA 1/2-sbsRNA binds the Alu element of the 3’-UTR region on the target gene in the Staufen 1 (STAU1)-mediated mRNA decay (SMD) pathway, thus forming a functional SBS. This SBS site transactivates STAU1 and mediates the binding of STAU1 to mRNA, recruits the binding of UPF1 protein to STAU1, and leads to the degradation of mRNA (Figure 7A). This is a novel mRNA degradation mechanism that induces protein binding to mRNA and regulates mRNA degradation [93]. The RNA binding protein STAU1 interacts with the Staufen 1 protein binding site in the 3’-UTR of mRNA, thus mediating the SMD degradation pathway. lncRNA containing a short interspersed element (SINE) sequence in mice can bind the Alu element in the 3’-UTR of mRNA and recruit the STAU1 protein to initiate the SMD degradation pathway. SMD degradation pathway also occurs in mouse cells, and mouse 1/2-sbsRNA (m1/2-sbsRNA)-triggered SMD regulates myogenesis of C2C12 cells. The m1/2-sbsRNA lncRNA decreases Cdc6 and Traf6 protein levels in C2C12 cells by binding the 3’ UTR region of *Traf6* and *Cdc6* mRNA, thus resulting in degradation of *Cdc6* and *Traf6* mRNA (Figure 7A) [94].

The lncRNA maternal expression gene 3 (MEG3), a maternally imprinted gene in the Dlk1-Gtl2 locus on chromosome 14q32.3 in human, is a tumor suppressor [95]. The lncRNA MEG3 plays a regulatory role in insulin resistance and liver fibrosis. Recently, RNA pull-down and LC-MS assays have revealed that lncRNA MEG3 binds polypyrimidine tract-binding protein 1 (PTBP1), which belongs to the hnRNPs subfamily and controls almost all aspects of mRNA metabolism [96]. RIP assay results have indicated that the PTBP1 protein binds the CDS region of small heterodimer partner (SHP) mRNA. Overexpressed PTBP1 or MEG3 promotes SHP mRNA decay. Down-regulation of SHP mRNA induced by MEG activates the expression of target genes *Cyp7a1* and *Cyp8b1*, thus leading to biliary stasis liver damage, destruction of bile acid homeostasis, elevated liver enzymes, including bile acid synthetase, and metabolic gene imbalance. Additionally, SHP inhibits the expression of MEG3 through cAMP response element-binding protein (CREB)-dependent feedback regulation (Figure 7B) [97].

### 5.4. lncRNAs Regulate RNA Stability

The stability of mRNA is a balance between the synthesis and degradation of the required RNA in the processes of gene transcription and translation. The regulation of mRNA stability occurs primarily through the degradation of various recognition proteins and nucleases. In vivo, the stability of mRNA is associated with the existence of specific binding proteins. Recent studies have reported that lncRNAs can also participate in regulating the stability of mRNA post-transcriptionally (Figure 1L). The lncRNA MACC1-AS1 is the antisense transcript of MACC1, an oncogene in gastric cancer (GC). LncRNA MACC1-AS1 is highly expressed in GC tissues and facilitates GC cell proliferation and viability under metabolic stress. The expression of MACC1-AS1 and MACC1 is positively correlated. Bioinformatic analysis has indicated that MACC1-AS1 includes a binding site for MACC1 mRNA. Further experiments have shown that MACC1-AS1 directly binds MACC1 through the binding site, thus increasing its stability and expression. AMPK plays an important role in regulating mRNA stability, especially under stress conditions [98]. Crosslinking-immunprecipitation and high-throughput sequencing (CLIP-seq) has revealed the interaction of MACC1 mRNA with the RNA binding protein Lin28. MACC1-AS promotes the distribution of Lin28 from the nucleus to the cytoplasm, and phosphorylated AMPK is increased in both the cytoplasm and nucleus. Phosphorylated AMPK enhances the translocation of Lin28. AMPK activation and subsequent Lin28 translocation play synergistic roles in regulating MACC1 mRNA stability (Figure 7C) [99].

Additionally, Wang et al. have identified a lncRNA *Sirt1* antisense transcript (Sirt1 AS) transcribed from the Sirt1 antisense strand in myogenesis. *Sirt1* AS lncRNA overexpression promotes myoblast proliferation but inhibits differentiation. miR-34a targets *Sirt1* mRNA, and the expression of miR-34a is negatively correlated with the expression of Sirt1 and *Sirt1* AS. The lncRNA *Sirt1* AS alleviates the inhibition of *Sirt1* expression and rescues the expression of proliferating genes by inhibiting the expression of miRNA-34a. *Srit1* AS lncRNA interacts with *Sirt1* mRNA, forming an RNA duplex, thus improving the stability of *Sirt1* through competition with miRNA-34a in the 3’-UTR region of the *Sirt1* gene and thereby promoting the proliferation and inhibiting the differentiation of C2C12 cells (Figure 7D) [100].

### 5.5. lncRNAs Encode Peptides

lncRNAs were previously defined as RNAs that do not encode proteins, but as research progressed, some lncRNAs were found not only to translate micropeptides (Figure 1M), but also to use these encoded micropeptides to perform biological functions. A skeletal muscle-associated lncRNA (LINC00948 in humans and AK009351 in mice) contains a short 138 nucleotide ORF and has the potential to encode 46 highly conserved amino acids. The micropeptide encoded by the lncRNA is conserved in vertebrates and is named myoregulin (MLN). MLN is expressed in almost all skeletal muscles and encodes a transmembrane alpha helix. MLN shows a strong structural resemblance to phosphoprotein and sarcolipin, and their transmembrane regions contain many of the same conserved residues. The function of MLN is similar to that of phosphoprotein and sarcolipin, through acting directly on the sarcoplasmic reticulum Ca^2+^-ATPase and preventing Ca^2+^ from entering the sarcoplasmic reticulum (Figure 7E). Deletion of the mouse MLN gene enhances the efficiency of skeletal muscle delivery to Ca^2+^ and improves exercise capacity [101].

Recently, researchers have found a polypeptide consisting of 90 amino acids (ORF1) encoded by the lncRNA LINC00961, which is highly expressed in skeletal muscle in both humans and mice. Knockout of LINC00961 in HeLa cells results in increased activation of mechanistic target of rapamycin complex 1 (mTORC1) under amino acid starvation and stimulation. The polypeptide, named ‘small regulatory polypeptide of amino acid response’ (SPAR) is conserved in both humans and mice, and localizes to the late endosome/lysosome. Further experiments have revealed that SPAR interacts with subunits of the v-ATPase proton pump, but it has no effect on v-ATPase proton pump activity. The v-ATPase complex interacts with the Ragulator complex at the lysosome, thereby regulating the activation of mechanistic target of rapamycin 1 (mTORC1) under amino acid stimulation [102]. SPAR affects the activity of mTORC1 and inhibits the recruitment of mTORC1 to lysosomes, primarily upstream of the Rags and Ragulator complexes, and at the level of v-ATPase (Figure 7F). The expression of the polypeptide encoding lncRNA LINC00961 is inhibited when skeletal muscle is injured. Using CRISPR/Cas9 technology to knockout only the polypeptide while retaining the gene of the host lncRNA, researchers have found that SPAR can decrease the activity of mTORC1 and promote muscle regeneration [103].

### 5.6. lncRNAs Are Involved in Protein Relocalization

LncRNAs regulate proteins in various ways, one of which is regulation of protein localization (Figure 1O). Rhizobium bacteria induce the formation of nodules on the roots of leguminous plants. *GmENOD40* is an lncRNA that is specifically expressed in legumes, is associated with symbiotic nitrogen-fixation nodule formation, and can encode short peptides. This lncRNA has a complex expression pattern during the development of soybean nodules, and studies have shown that *ENOD40* peptide may have a transport function and participate in the control of sucrose use in nitrogen-fixing nodules [104,105]. Homologous genes of *GmENOD40* have been identified in *Medicago truncatula*. *MtENOD40* shows homology to a soybean early nodulin gene, *GmENOD40*. *MtENOD40* localizes in the cytoplasm of cells in the nodule primordium and has a role in plant growth control and differentiation [106]. *ENOD40* is rapidly induced by rhizobia in the root pericycle and in the dividing cortical cells of the primordial nodules [107] and other non-symbiotic organs. Yeast three-hybrid experiments have shown that *MtENOD40* interacts with the continuously expressed RNA binding protein MtRBP1 in *Medicago truncatula*. MtRBP1 is located in the nuclear speckles of plant cells, but it is exported to the cytoplasm during nodule development in *ENOD40*-expressing cells. Transient expression experiments have shown that *ENOD40* RNA is directly involved in the relocalization of MtRBP1 to cytoplasmic granules. These results suggest that *MtENOD40* plays a major role in guiding MtRBP1 from the nucleus to the cytoplasm (Figure 7G) [108].

### 5.7. lncRNAs Regulate RNA Methylation of mRNA

N^6^-methyladenosine (m^6^A) is an RNA methylation modification on the sixth nitrogen atom of adenine in RNA molecules. DNA and histone methylation primarily play roles at the transcriptional level, whereas reversible RNA methylation primarily regulates gene expression at the post-transcriptional level. m^6^A is one of the most common post-transcriptional modifications of eukaryotic RNA, and it plays important roles in accelerating the metabolism and translation of RNA, as well as in cell differentiation, embryonic development, and stress responses. Various studies have shown that m^6^A is found in many species, accounting for 80% of RNA methylation modifications. N^6^-methyladenosine is distributed not only in mRNA but also in many non-coding RNAs, including circRNAs and lncRNAs [109]. m^6^A methylation modification is reversible because of the concomitant action of methyltransferases (writers), demethylases (erasers), and proteins able to recognize methylated RNA (readers) [110]. Previous studies have revealed the important role of m^6^A in mRNA modification; studies of lncRNA-regulated m^6^A modification of mRNA have been reported in recent years (Figure 1N), primarily in animals.

The RNA demethylase ALKBH5 is associated with poor prognosis of malignant glioma and promotes cancer cell proliferation. m^6^A-seq and transcriptome sequencing have been carried out on ALKBH5 knockout cells, thus identifying the target gene FOXM1. ALKBH5 colocalizes and interacts with FOXM1 nascent transcripts, thereby maintaining FOXM1 nascent transcript, primarily the 3′ UTR, demethylated. Given that the RNA binding protein HuR binds preferentially the unmethylated 3’-UTR of FOXM1, ALKBH5 activity affects the binding of HuR to FOXM1 and increases the stability of its transcript. LncRNA FOXM1-AS is opposite from the transcription direction of FOXM1 and complementary to the last exon of FOXM1 mRNA with 457 nucleotides; it *cis*-regulates the expression of FOXM1 and co-localizes with FOXM1 and ALKBH5. FOXM1-AS interacts with FOXM1 nascent transcripts and ALKBH5. FOXM1-AS cooperates with ALKBH5 in regulating expression of FOXM1 nascent transcripts through promoting the binding of HuR to FOXM1, and it plays an important role in glioblastoma stem-like cells (GSC) tumorigenesis (Figure 7H) [111].

## 6. lncRNAs Are Involved in Translational Regulation

Genetic information is transmitted from DNA to RNA, and then from RNA to protein, in the process of transcription and translation of genetic information. Gene mRNAs perform biological functions in various life activities through translation into proteins. lncRNAs not only participate in DNA and RNA regulation at the transcriptional and post-transcriptional levels, but also inhibit or promote gene expression at the translational level (Figure 1Q–P).

**Figure 8:**
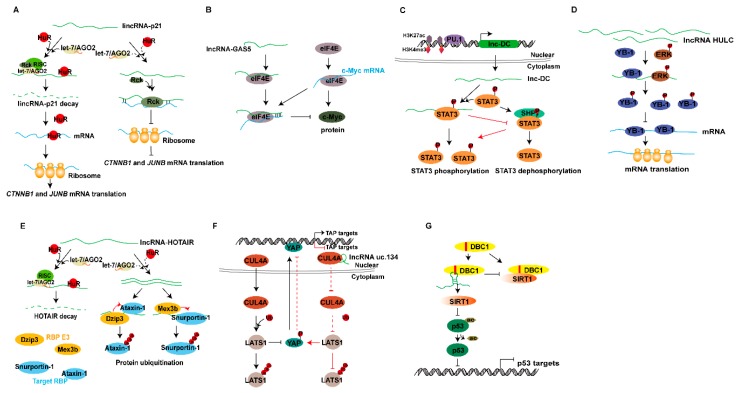
lncRNAs participate in regulating translation and post-translational modification of proteins. (**A**,**B**) Mechanism of lncRNA regulation of mRNA translation. (**A**) HuR and let-7/Ago2 bind to lincRNA-p21, which negatively regulates lincRNA-p21 and promotes lincRNA-p21 decay, thereby facilitating target β-catenin (*CTNNB1*) and JunB (*JUNB*) mRNA translation. When HuR is absent, Rck promotes the association of lincRNA-p21 with *CTNNB1* and *JUNB* mRNAs, thus inhibiting their translation. (**B**) The translation initiation complex eIF4E binds c-Myc mRNA and promotes its translation. lncRNA growth arrest-specific 5 (GAS5) binds eIF4E and c-Myc mRNA, and GAS5 regulates c-Myc translation through lncRNA–RNA interaction and cooperates with eIF4E. (**C**,**D**) Mechanism model of how lncRNAs regulate protein phosphorylation. (**C**) Histone H3K4me3 and H3K27ac modifications and the transcription factor PU.1 directly activate lnc-DC transcription. lnc-DC and SHP1 bind signal transducer and activator of transcription 3 (STAT3) protein; lnc-DC binds the C-terminus of STAT3, prevents dephosphorylation of STAT3 Tyr705 by SHP1, and activates transcriptional activity. (**D**) lncRNA HULC interacts with extracellular signal-regulated kinase (ERK) and Y-box protein-1 (YB-1) and promotes phosphorylation of YB-1, thereby reducing the interaction of certain oncogenic mRNAs with YB-1 and enhancing mRNA translation. (**E**,**F**) Models for lncRNA regulation of protein ubiquitination. (**E**) HuR interacts with HOX antisense intergenic RNA (HOTAIR) and promotes its degradation by recruiting the let7–Ago2 complex. When HuR is silent, HOTAIR acts as a scaffold for Mex3b and Snurportin-1, Dzip3 and Ataxin-1, respectively, thereby promoting Snurportin-1 and Ataxin-1 protein ubiquitination and degradation. (**F**) Layer one (red line): lncRNA uc.134 directly binds to the E3 ubiquitin ligase CUL4A protein and inhibits its translocation from the nucleus to the cytoplasm, thus decreasing LATS1 ubiquitination, promoting phosphorylation of YAP, and silencing YAP target genes expression. Layer one (black line): when lncRNA uc.134 is absent, CUL4A protein enters the cytoplasm from the nucleus and activates the ubiquitination of LATS1, which blocks the phosphorylation of YAP and promotes YAP target gene expression. (**G**) A model for lncRNA regulation of protein acetylation. lncRNA MALAT1 interacts with depleted in breast cancer 1 (DBC1), inhibits binding between DBC1 and SIRT1, and enhances the deacetylation activity of SIRT1, which promotes deacetylation of p53, thus inhibiting the function of p53. ‘→’ indicates facilitation and activation, ‘⟞’ indicates repression.

LincRNA-p21 was initially identified as a transcriptional repressor in p53 gene-dependent transcriptional process. p53 is an important tumor suppressor gene that maintains genomic integrity, and incRNA-p21 was shown to affect p53-dependent apoptosis through the binding to heterogeneous nuclear ribonucleoprotein-K [112]. Additionally, lincRNA-p21 was shown to function also as a translational modulator and an important player in the function of the RNA-binding protein HuR. lincRNA-p21 interacts with target β-catenin (*CTNNB1*) and JunB (*JUNB*) mRNAs and inhibits their translation efficiency in a process that requires the translational repressor Rck. HuR and let-7/Ago2 synergistically inhibit the expression of lincRNA-p21 by inducing its degradation. The degradation of lincRNA-p21 promotes binding of HuR to the target *CTNNB1* and *JUNB* mRNAs and subsequent translation of *CTNNB1* and *JUNB* mRNAs, thus increasing the levels of these proteins. In the case of decreased HuR levels, lincRNA-p21 accumulates in human cervical cancer HeLa cells, and its binding to *JUNB* and *CTNNB1* mRNAs is associated with a selective reduction in their translation (Figure 8A) [113].

Growth arrest-specific 5 (GAS5), an lncRNA plays an important role in regulating mammalian cell apoptosis and cell population growth, is frequently inhibited in many cancers. Studies have indicated that lncRNA GAS5 interacts with the translation initiation machinery and regulates mRNA translation. GAS5 contains two novel RNA-binding motifs that bind the translation initiation complex eIF4E (eukaryotic initiation factor) at the initiation of translation, and play a role in the translation regulation. Overexpression of in vitro transcribed GAS5 suppresses c-Myc protein without affecting c-Myc mRNA, whereas GAS5 siRNA enhances the c-Myc protein level, thus demonstrating that GAS5 specifically regulates c-Myc protein expression level. The mechanism of the increase in c-Myc protein is that the binding of c-Myc mRNA to polysomes is enhanced without affecting the stability of the protein. Interestingly, GAS5 binds to c-Myc mRNA, thus suggesting that GAS5 regulates c-Myc translation through lncRNA–RNA interaction. Further analyses have indicated that GAS5 interacts with eIF4E in the regulation of c-Myc translation (Figure 8B) [114].

## 7. LncRNAs Are Involved in Post-translational Modification

Post-translational modification of proteins is an important part of proteomics. Post-translational modification of proteins changes their spatial conformation, activity, stability and interaction with other molecules, and consequently participates in regulating diverse life activities in the body. Post-translational modification of proteins is a complex process. Currently, more than 20 modification types are known in eukaryotes, including phosphorylation, acetylation, ubiquitination, glycosylation, and SUMOylation [115]. lncRNAs are involved in various post-translational modifications of proteins, mainly protein phosphorylation, ubiquitination, and acetylation, thereby regulating protein degradation or formation, and affecting protein expression levels (Figure 1R). Next, we introduce the mechanisms through which lncRNAs regulate protein phosphorylation, ubiquitination, and acetylation.

### 7.1. lncRNAs Are Involved in Regulating Protein Phosphorylation

Phosphorylation, the most extensive covalent modification of protein after translation, plays an important role in cell signal transduction. Protein phosphorylation occurs mainly on serine and tyrosine residues, and is regulated by some protein kinases. lncRNAs have been found to regulate protein phosphorylation. lncRNA lnc-DC is solely expressed in human conventional dendritic cells (DCs) and mediates DC differentiation. Histone H3K4me3 and H3K27ac modifications and the transcription factor PU.1 directly activate lnc-DC transcription. RNA fluorescence in situ hybridization (RNA FISH) has indicated that lnc-DC is localized in the cytoplasm. RNA pull-down and RIP assays have confirmed that lnc-DC interacts with signal transducer and activator of transcription 3 (STAT3) protein; RNA FISH has further verified that lnc-DC colocalizes with STAT3 in the cytoplasm. lnc-DC enhances STAT3 phosphorylation on Tyr-705 and participates in its post-translational modification. SHP1 is a protein tyrosine phosphatase that promotes dephosphorylation of tyrosine. Knockout of lnc-DC promotes the binding of SHP1 and STAT3, thereby enhancing the dephosphorylation of STAT3, whereas overexpression of lnc-DC attenuates their binding, preventing dephosphorylation of STAT3 Tyr705, and activating downstream transcriptional activity (Figure 8C) [116]. Another aspirin-induced upregulated lncRNA, OLA1P2, which is found in human colorectal cancer, is transcriptionally activated by the transcription factor FOXD3 (forkhead box D3). Because OLA1P2 has the same conserved STAT3 transcriptional response element as the STAT3 target gene, it directly interacts with STAT3 and inhibits its phosphorylation. The aspirin-FOXD3-OLA1P2-STAT3 axis has a significant anticancer effect [117].

lncRNA HULC, the first lncRNA identified in liver cancer, enhances the cell malignant phenotype and cell proliferation by promoting G1/S transformation. RNA pull-down, RIP, deletion-mapping, and colocalization analyses have indicated that HULC specifically interacts with YB-1 (Y-box protein-1). Extracellular signal-regulated kinase (ERK) protein kinase has been shown to phosphorylate YB-1, and RNA pull-down experiments have shown that HULC interacts with ERK. Elevated expression of HULC promotes phosphorylation of YB-1 by activating the ERK pathway, thereby reducing the interaction of certain oncogenic mRNAs with YB-1, such as cyclin D1, cyclin E1, and MMP11, and accelerating the translation of these mRNAs during tumorigenesis and promoting cell proliferation (Figure 8D) [118].

### 7.2. lncRNAs Are Involved in Regulating Protein Ubiquitinlation

Protein ubiquitination is a common form of post-translational modification, which regulates various protein substrates in different cellular pathways. Ubiquitin labels proteins to be degraded in proteasomes, a process requiring three enzymes: ubiquitin-activating enzyme (E1), ubiquitin-binding enzyme (E2), and ubiquitin protein ligase (E3). lncRNAs have a variety of functions, such as regulating protein ubiquitination and affecting protein activity. lncRNA HOTAIR (HOX antisense intergenic RNA) is involved in various regulatory pathways, including regulating the ubiquitination of proteins. HuR interacts with HOTAIR, thereby promoting its degradation by recruiting the let7–Ago2 complex. RIP experiments have indicated that HOTAIR specifically binds to two E3 ubiquitin ligases Mex3b and Dzip3 and their respective substrates Snurportin-1 and Ataxin-1. HOTAIR is mainly used as a scaffold molecule that, by bringing together ubiquitin ligases with their substrates, increases substrate degradation. Accordingly, overexpression of HOTAIR promotes ubiquitination and protein degradation of Snurportin-1 and Ataxin-1 (Figure 8E) [119].

lncRNA uc.134 is an ultraconserved lncRNA, and its expression is significantly decreased in highly aggressive HCC cells. Gain-of-function and loss-of-function studies in vitro and in vivo have indicated that the lncRNA uc.134 inhibits HCC cell proliferation, invasion, and metastasis. RNA pull-down, RIP, and western blot (WB) assays have confirmed that lncRNA uc.134 directly interacts with the E3 ubiquitin ligase CUL4A. lncRNA uc.134 affects the localization of CUL4A, and overexpression of uc.134 promotes the accumulation of CUL4A protein in the nucleus, whereas knockout of uc.134 increases CUL4A protein levels in the cytoplasm. LATS1 protein contains an N-terminal ubiquitin-binding domain, and CUL4A activates ubiquitination and degradation of LATS1. Ubiquitination of LATS1 blocks its kinase activity in phosphorylating YAP, primarily at Ser127 [120]. By directly binding to CUL4A, lncRNA uc.134 suppresses its translocation from the nucleus to the cytoplasm, reduces ubiquitination and degradation of LATS1, activates Hippo kinase signaling, and promotes phosphorylation of the LATS1 target gene YAP, thereby sentencing YAP target gene expression (Figure 8F) [121].

### 7.3. lncRNAs Are Involved in Regulating Protein Acetylation

We have previously mentioned the regulation of histone acetylation by lncRNA. In addition to histone acetylation, non-histone acetylation occurs; dynamic changes in protein acetylation and deacetylation are regulated by acetyltransferase and deacetylase. Non-histone acetylation also plays an important role in life processes [122]. Similarly, lncRNAs are also involved in the regulation of protein acetylation, thus affecting protein activity. lncRNA MALAT1 is involved in many aspects of cellular processes. To further elucidate the mechanism of interaction between lncRNA MALAT1 and proteins, proteins interacting with MALAT1 have been identified by quantitative proteomics. The depleted in breast cancer 1 (DBC1) was identified through stable isotope labeling by amino acids in cell culture (SILAC) quantitative proteomic analysis, RIP, RNA pull-down, and WB assays, thus confirming that MALAT1 physically interacts with the aa120-280 region of DBC1. DBC1 contains five major functional domains, one of which is the leucine zipper (LZ) domain, which binds to the catalytic domain of the protein deacetylase SIRT1, and inhibits SIRT1 deacetylation activity [123]. Knockout and overexpression of MALAT1 increases and inhibits the binding of DBC1 to SIRT1, thereby regulating the activity of SIRT. SIRT1 promotes deacetylation of p53 [124], and knocking out MALAT1 increases the acetylation of p53. Co-transfection experiments have shown that MALAT1 regulates the acetylation of p53 by interacting with DBC1. MALAT1 promotes deacetylation of p53 and inhibits the transcriptional activity of p53 by competitively binding to DBC1 with SIRT1, thereby inhibiting the function of p53 and promoting cell proliferation (Figure 8G) [125].

## 8. Conclusions and Perspectives

lncRNAs account for 80% of ncRNA, have no protein-coding function, and play roles as RNA molecules. lncRNAs participate in regulation at the epigenetic, transcriptional, post-transcriptional, translational, and post-translational levels in the form of RNA. In addition, recent studies have found that lncRNAs can encode polypeptides and regulate gene expression in the form of polypeptides. Studies have demonstrated multiple mechanisms of lncRNAs in animals, such as lncRNA-encoded peptides and interaction with DNA or RNA methylases and demethylases; however, the mechanism of action of lncRNAs to regulate RNA methylation of mRNA and protein expression have not been reported in plants. Although plant lncRNAs involved in these functions have not been reported yet, we have reason to believe that many of the unknown functions of lncRNAs will be realized through these modes. These mechanisms of lncRNA action in plants can provide directions for future research, and more functions of plant lncRNAs can be determined.

lncRNAs do not function in a single manner or function alone; instead, they interact with other genes and proteins, and their functions are complex. On the basis of the summary of the above mechanisms, lncRNAs primarily interact with other molecules and directly or indirectly regulate gene transcription and translation, not only through simple single regulatory pathways but also through participating at multiple regulatory levels. The field of lncRNAs research is also expanding rapidly thanks to developing technologies such as CRISPR/Cas9, RNA pull-down, RIP, ChIP, and ChIRP that have helped in understanding the functions and mechanisms of action of lncRNAs. Recently, these experimental techniques have been applied in plants, thus greatly aiding in the study of the functions and mechanisms of lncRNAs, as well as in promoting the rapid development of plant lncRNA research. By combining these technologies with other future technologies, more attractive and unique features and functions of lncRNAs will certainly be found. Obviously, there is no universal function of lncRNAs, and only through specific case studies can meaningful understanding be obtained. When we study lncRNAs, we cannot classify them as independent actors, as this would limit our thinking and understanding of their functions and mechanisms.
